# Brain-aging related protein expression and imaging characteristics of mice exposed to chronic hypoxia at high altitude

**DOI:** 10.3389/fnagi.2023.1268230

**Published:** 2023-10-02

**Authors:** Yaxin Cao, Shundao Cao, Ri-Li Ge, Haihua Bao, Yalin Mou, Weizhong Ji

**Affiliations:** ^1^Tangdu Hospital of Air Force Military Medical University, Xi’an, China; ^2^Department of Neurology, Xi’an No. 1 Hospital, Xi’an, China; ^3^Research Center for High Altitude Medicine, Qinghai University, Xining, China; ^4^Department of Medical Imaging Center, Qinghai University Affiliated Hospital, Xining, China; ^5^Qinghai Provincial People’s Hospital, Xining, China

**Keywords:** high altitude, hypoxia, neuron apoptosis, brain aging, magnetic resonance imaging, brain structure

## Abstract

**Objective:**

To determine changes in protein expression related to brain aging and imaging features in mice after chronic hypoxia exposure at high altitude.

**Method:**

A total of 24 healthy 4-week-old mice were randomly divided into high altitude hypoxia (HH) and plain control (PC) groups (*n* = 8 per group). HH mice were transported from Xi’an (450 m above sea level) to Maduo (4,300 m above sea level) while PC mice were raised in Xi’an. After 6 months, 7.0T magnetic resonance imaging (MRI) was performed. All mice completed T2-weighted imaging (T2WI), diffusion tensor imaging (DTI), resting-state functional MRI (rs-fMRI), arterial spin labeling (ASL), and magnetic resonance angiography (MRA) examinations. Next, brain slices were prepared and Nissl staining was used to observe morphological changes in neurons. Ultrastructural changes in neurons were observed by transmission electron microscopy. Expression changes of Caspase-3, klotho, P16, P21, and P53 at the gene and protein levels were detected by real-time PCR (RT-PCR) and Western blot.

**Results:**

The number of neuronal Nissl bodies in the hippocampus and frontal cortex was significantly decreased in the HH group compared to the PC group. Some hippocampal and frontal cortical neurons were apoptotic, the nuclei were wrinkled, chromatin was aggregated, and most mitochondria were mildly swollen (crista lysis, fracture). Compared with the PC group, the HH group showed elevated expression of caspase-3 mRNA, P16 mRNA, P21 mRNA, and P53 mRNA in the hippocampus and frontal cortex. Expression of Klotho mRNA in the frontal cortex was also significantly decreased. Western blot results showed that caspase-3 protein expression in the hippocampus and frontal cortex of the HH group was increased compared with the PC group. Moreover, there was decreased Klotho protein expression and significantly increased P-P53 protein expression. Compared with the PC group, expression of P16 protein in the frontal cortex of the HH group was increased and the gray matter (GM) volume in the left visceral area, left caudate nucleus, and left piriform cortex was decreased. Furthermore, the amplitude of low frequency fluctuation was decreased in the left posterior nongranular insular lobe, right small cell reticular nucleus, left flocculus, left accessory flocculus, and left primary auditory area, but increased in the GM layer of the left superior colliculus. Regional homogeneity was decreased in the left and right olfactory regions, but increased in the left bed nucleus. After exposure to high altitude, functional connectivity (FC) between the bilateral caudate nucleus and thalamus, corpus callosum, cingulate gyrus, anterior limbic cortex, globus pallidus, and hippocampus was weakened. FC between the right caudate nucleus and hypothalamus and entorhinal cortex was also weakened. The fractional anisotropy value of the left hippocampus was decreased in the HH group. Compared with the PC group, the HH group showed significantly increased inner diameters of the bilateral common carotid artery and left internal carotid artery. The cerebral blood flow values of the bilateral cortex and bilateral hippocampus in the HH group did not change significantly.

**Conclusion:**

Taken together, our findings show that chronic hypoxia exposure at high altitude may promote neuronal apoptosis and abnormal expression of related proteins, changing the structure and function of brain. These changes may contribute to brain aging.

## Introduction

Both population studies and animal experiments have demonstrated that acute and long-term chronic hypoxia can cause brain functional injury (Zhang et al., 2013; Xu et al., 2021). The hippocampus and frontal cortex are among the most sensitive brain areas to hypoxia. Chronic hypoxia exposure at high altitude has been shown to cause increased apoptosis of neurons in the hippocampus and frontal cortex of rats and lead to impaired learning and memory (Ji et al., 2021a,b). Maiti et al. (2008) observed neuronal apoptosis in several brain regions including the cortex, hippocampus, and striatum of rats after exposure to high altitude, which may be related to cognitive impairment at high altitude. Despite such findings, it remains unclear whether exposure to high altitude accelerates overall brain aging and abnormal expression of brain aging-related proteins. Prior studies have found that hypoxia may lead to premature aging of the body (Yeo, 2019), but there are also studies showing that intermittent hypoxia training can improve cerebrovascular function, thereby slowing down the decline of brain function (Manukhina et al., 2016). Thus, whether long-term chronic hypoxic environmental exposure promotes brain aging is currently controversial.

In recent years, magnetic resonance imaging (MRI) technology has been applied to study changes in human brain structure and function after exposure to high-altitude, low-oxygen environments (Chen et al., 2019; Lefferts et al., 2019). A study of high-altitude sojourners (Yan et al., 2010) found significant gray matter (GM) loss and volume shrinkage in several brain regions including the cortex, striatum, and hippocampus. Bao et al. (2022) found that, compared with the control group, the GM volume in the left inferior temporal gyrus, right middle temporal gyrus, right caudate nucleus, right insula lobe, and bilateral lentiform nucleus increased in the chronic hypoxia group, whereas the GM volume in the left middle occipital gyrus and left middle temporal gyrus decreased. Notably, the results of these two studies are inconsistent. As previous high-altitude brain MRI research mainly concentrated on high-altitude populations and plains populations or plains residents who migrated to high-altitude areas, the results could be affected by factors such as lifestyle and educational background. In addition, the MRI technology used in prior studies was relatively simple and unable to comprehensively evaluate structural changes in nerves, blood vessels, and blood flow in the brain. The establishment of animal models of high-altitude hypoxia exposure and the use of multimodal MRI technology along with morphological and molecular biological methods to evaluate changes in brain structure and related protein expression after hypoxia exposure can avoid the above issues to provide more comprehensive and objective results.

## Materials and methods

A total of 24 healthy male C57BL/6 mice were randomly divided into two groups: high-altitude hypoxia (HH) group and plain control (PC) group. The groups were reared for 6 months in Xi ‘an (450 m) or Madol (4,300 m). The two groups did not differ in their daily food and water consumption. The temperature of the animal room is controlled at 18°C–22°C, and the light time is 8 h. All mice were kept in cages with 4 mice per cage, kept clean and dry under natural light, changed bedding regularly, drank water freely, and fed with feed that met national standards. All operations are in accordance with the requirements of the Regulations on the Management of Experimental Animals and the scientific research ethics requirements of Qinghai Provincial People’s Hospital. After 6 months of living at the two altitudes, the groups underwent 7.0 T head MRI. T1-Weighted Imaging (T1WI), T2-Weighted imaging (T2WI), DTI, MRA, rs-fMRI and other imaging information were collected. Subsequently, fresh and perfused brain tissue samples were prepared for morphological and molecular biology analyses.

### Preparation for MRI

A PharmaScan 70/16 US 7.0T MRI scanning system for small animals (Bruker, Germany) and R510-22-16 animal anesthesia machine (Shenzhen Reward Life Technology Co., LTD.) were used for scanning. Anesthesia was induced with 30% oxygen and 3% isoflurane mixture gas with an airflow speed controlled at 3–4 mL /min for 2–3 min. The success of anesthesia was indicated when the mouse’s turning reflex disappeared. The mice were then fixed on the scanning bed in a prone position and anesthesia was maintained with 1.5% isoflurane. During the scanning process, a hot water circulation system was used to maintain the mouse’s body temperature to ensure a stable physiological state. The respiratory and heartbeat frequencies were monitored with respiration controlled at 40–60 times/min. A mouse head coil was used for the head positioning scan and T1WI, T2WI, DTI, MRA, rs-fMRI, and other imaging data were collected.

### MRI imaging parameters

The T1WI scanning parameters were as follows: echo time (TE): 8.00 ms, repetition time (TR): 750.00 ms, scanning field: 20 mm × 20 mm, image matrix: 256 × 256, and rotation angle: 90°. The T2WI scanning parameters were: TE: 24.5 ms, TR: 1500.00 ms, scanning field: 25 mm × 25 mm, image matrix: 256 × 256, and rotation angle: 90° for both methods, 24 images were collected for each sequence. The DTI scanning parameters were: TE: 27.620 ms, TR: 4000.00 ms, b value: 800 mm/s, image matrix: 128 × 128, and scanning field: 25 mm × 25 mm. The rs-FMRI scanning parameters were: TE: 22 ms, TR: 2500.00 ms, scanning field: 25 mm × 25 mm, image matrix: 128 × 128, and rotation angle: 90°.

### MRI data processing

VBM pretreatment was performed on the Matlab2014b platform using SPM12^1^ and DPABI^2^. This software package preprocesses the imaging data successively through data format conversion, voxel amplification, slice timing correction, head motion correction, organization segmentation, spatial standardization, and spatial smoothing and then calculates the related indexes.

For fMRI analysis preprocessing, the same software package was used to perform data format conversion, voxel amplification, slice timing correction, head motion correction, tissue segmentation, and spatial standardization. Subsequently, noise reduction was performed and the regional homogeneity (ReHo) index, amplitude of low-frequency fluctuation/fractional amplitude of low-frequency fluctuation (ALFF/fALFF) index and functional connectivity (FC) index were calculated.

### Analysis of mouse brain slices

Fresh mouse brain tissue slices were prepared after MRI scanning. The cervical spine position was determined, the cervical spine was severed, the foramen magnum was exposed, and the eye socket was severed. The skin on the top of the head was cut and the cranial nerves were separated from the brain tissue using vascular forceps. The brain tissue was removed and the cortex and hippocampus were separated. The tissue was then placed in cryostorage tubes (liquid nitrogen preservation).

To prepare postperfusion brain tissue samples after scanning, the skin was cut along both sides of the costal arch, the abdominal cavity was opened, the diaphragm and ribs were cut, and the heart was fully exposed. A puncture needle was inserted into the apex of the left ventricle and then into the aorta. The right auricle was cut, sterile saline was injected until the outflow was clarified, and paraformaldehyde was then injected. The cervical vertebrae were clamped, excess bone slices were removed, the foramen magnum was exposed, and the orbital sockets were clamped. The skin on the top of the head was cut and the cranial nerve and brain tissue was separated from the cerebellum using vascular forceps. The tissue was then removed and soaked in paraformaldehyde for 24 h.

### Nissl staining

For routine dewaxing, the sections were placed in 1% toluidine blue aqueous solution preheated to 50°C and dyed in a 56°C temperature box for 20 min. Next, the sections were washed with distilled water, soaked in 70% alcohol for 1 min, and treated with 95% alcohol differentiation. Anhydrous alcohol was used for rapid dehydration followed by treatment with transparent xylene and neutral gum seal. Finally, the sections were observed using a Pannoramic 250 microscope.

### Electron microscope staining

Three specimens were taken from each group. After anesthesia, brain tissue was removed by decapitation. Cortex and hippocampus tissues were quickly separated, prefixed with 3% glutaraldehyde, refixed with 1% osmium tetroxide, dehydrated by an acetone step, and embedded with Ep812. Semi-thin sections were stained with toluidine blue for optical positioning. Ultra-thin sections were obtained using a diamond knife and uranium acetate and lead citrate staining was performed. The ultrastructure of neurons in the hippocampus and cortex was observed using a JEM-1400 Flash transmission electron microscope.

### RT-PCR

Total RNA was extracted from mice hippocampus and cortex using the TRIzol one-step method, dissolved in DEPC water, and then stored at 4°C. Premier primer design software was used to design and screen specific primers for each gene. A two-step RT-PCR assay, genomic DNA removal, and reverse transcription reaction was then performed. The qRT-PCR amplification reaction system (20.0 μL) was constructed. The following mixture was prepared in q-PCR tubes: 2 × Real PCR EasyTM Mix-SYBR 10.0 μL, forward primer (10 μM) 0.8 μL, reverse primer (10 μM) 0.8 μL, template DNA 2.0 μL, ddH2O 6.4 μL to a total volume of 20.0 μL. The qRT-PCR reaction conditions were: predenaturation at 95°C for 30 s, denaturation at 95°C for 5 s, annealing at 55°C for 30 s, full extension at 72°C, and fluorescence collection for 30 s for a total of 45 cycles. Thermo Scientific PikoReal software (Thermo Company) was used to analyze the cycle threshold (CT) values of each test sample. The relative mRNA expression level of each target gene was calculated using the 2^−△△CT^ method (Table 1).

**Table 1:** Primers and base sequences for RT-PCR detection.

Primer name	Upstream	Downstream
ACTB	catcactattggcaacgagcggttcc	acgcagctcagtaacagtccgccta
Caspase-3	gaaactcttcatcattcaggcc	gcgagtgagaatgtgcataaat
KLotho	cagcgatagttacaacaacgtc	gatatggagaagcggtagtgg
P16	tcaagacatcgtgcgatatttg	ttagctctgctcttgggattg
P21	atgtccaatcctggtgatgtc	gaagtcaaagttccaccgttc
P53	tggaaggaaatttgtatcccga	gtggatggtggtatactcagag

### Western blots

The total protein of the hippocampus and cortex was extracted using the Radio Immunoprecipitation Assay (RIPA) method. After quantification of BCA protein, SDS-polyacrylamide (PAGE) gel electrophoresis buffer solution was added for each group to an equal concentration and equal weight. Transmembrane, closure, incubation antibody, and chemiluminescent solution (ECL) exposure development were performed. GIS control software was used to scan the strips for exposure and to analyze the gray values. The results were determined by the relative expression of the target protein as follows: target protein relative expression = target protein integral optical density (IOD)/intrinsic integral optical density (IOD).

### Statistical analysis

SPSS 22.0 software (IBM, USA) was used for statistical analysis. First, a normality test was performed. Data conforming to a normal distribution are reported as the mean ± standard deviation. When the *p*-value was <0.05, the difference between groups was considered to be statistically significant.

For imaging results, the statistical modules provided by DPABI and SPM12 were used to perform multiple two-sample *t*-tests for gray matter (GM) volume, ReHo value, ALFF, fALFF, and FC results between the two groups. Family-wise error (few) correction using DPABI was applied for multiple comparison correction. The statistical significance criteria were a voxel level *p* < 0.001 and cluster level *p* < 0.05.

## Results

### Morphological changes

**Figure 1:**
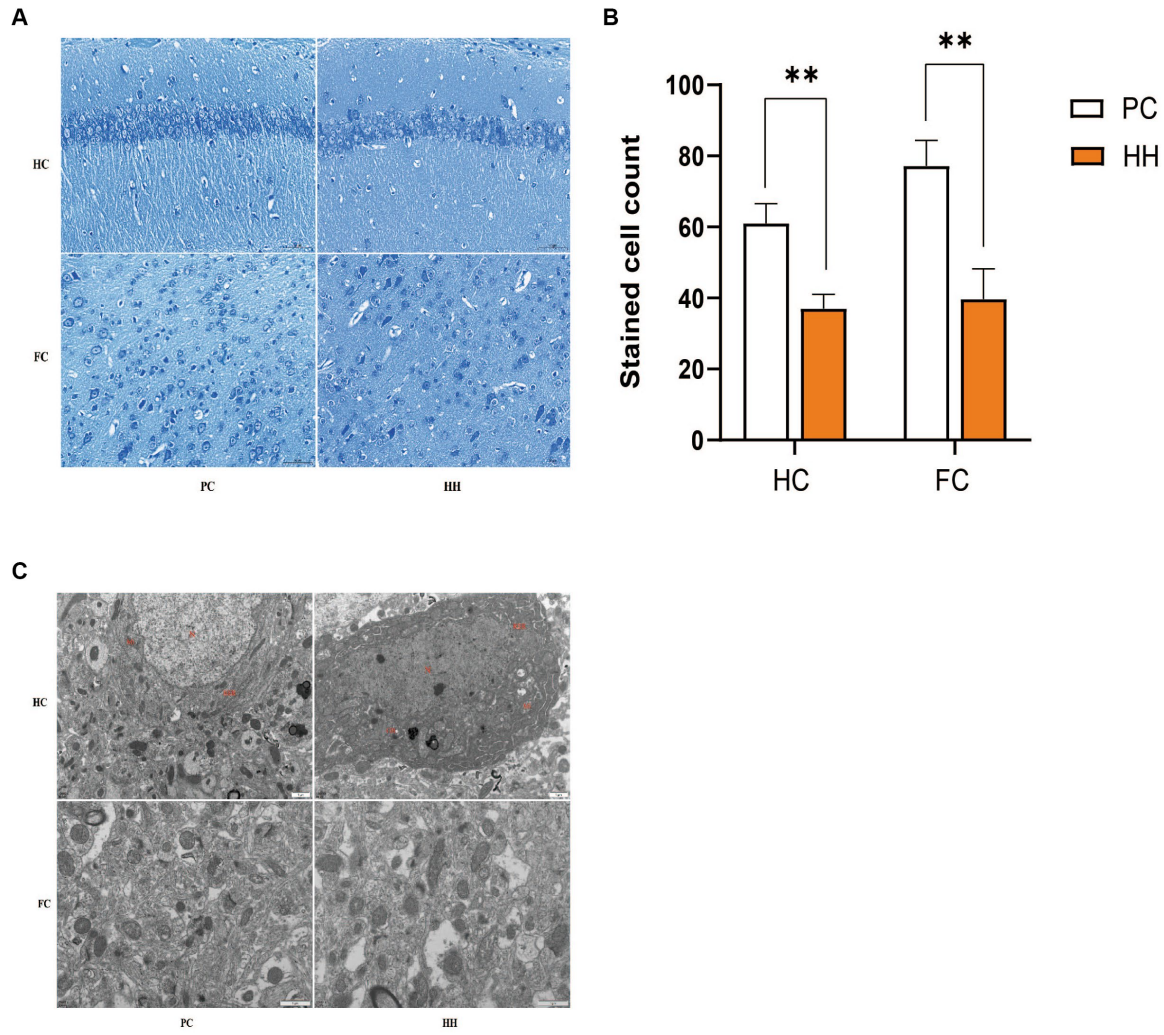
Comparison of the number of Nissl bodies in hippocampal and cortical neurons **(A,B)** and ultrastructure **(C)** observed at 12000× magnification between the two groups **p <0.01.

Compared with PC group, the number of neuronal Nishi bodies in the hippocampus and cortex of the HH group were significantly decreased (*p* < 0.01), suggesting that high altitude exposure damaged the neurons (Figures 1A,B). Apoptosis of neurons in the HH group was more obvious and the cell nuclei were wrinkled, the chromatin was gathered, the density of cytoplasmic and nuclear electrons increased, and most mitochondria showed mild swelling (Figure 1C).

### Changes in gene expression of brain aging-related proteins

**Figure 2:**
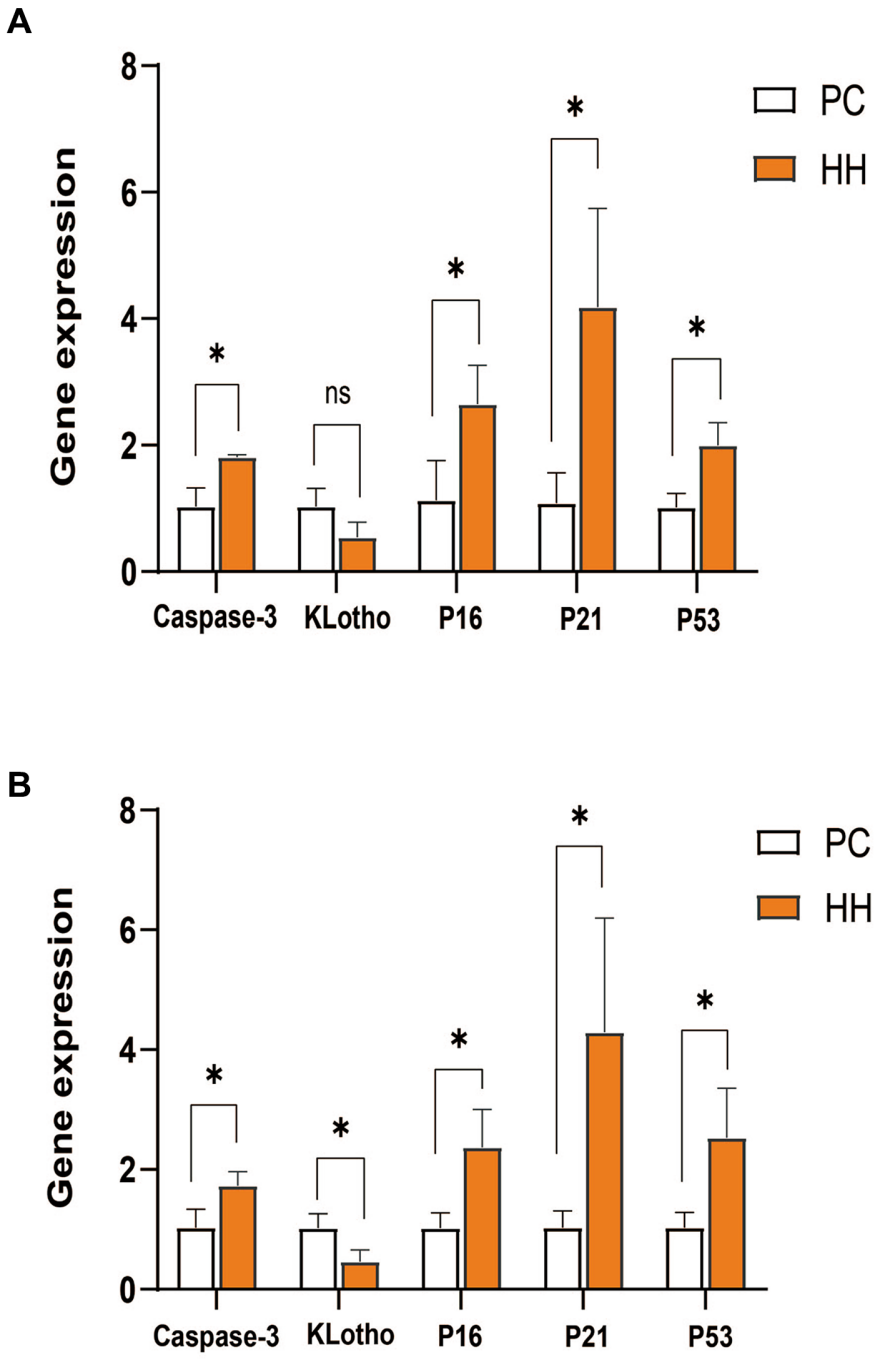
Comparison of mRNA expression of related genes in the hippocampus and frontal cortex of mice in the two groups. **(A)** Expression changes of various proteins in the hippocampus. **(B)** Expression changes of various proteins in the frontal cortex *p <0.05.

Compared with PC group, mRNA expression of caspase-3, P16, P21, and P53 in the hippocampus of the HH group was significantly increased (*p* < 0.05). There was no significant group difference in Klotho mRNA expression (*p* > 0.05; Figure 2A). Compared with the PC group, mRNA expression of caspase-3, P16, P21 and P53 in cortical tissues of the HH group was significantly increased (*p* < 0.05), while KLotho mRNA expression was significantly decreased (*p* < 0.05; Figure 2B).

### Changes in brain aging-related protein expression

**Figure 3:**
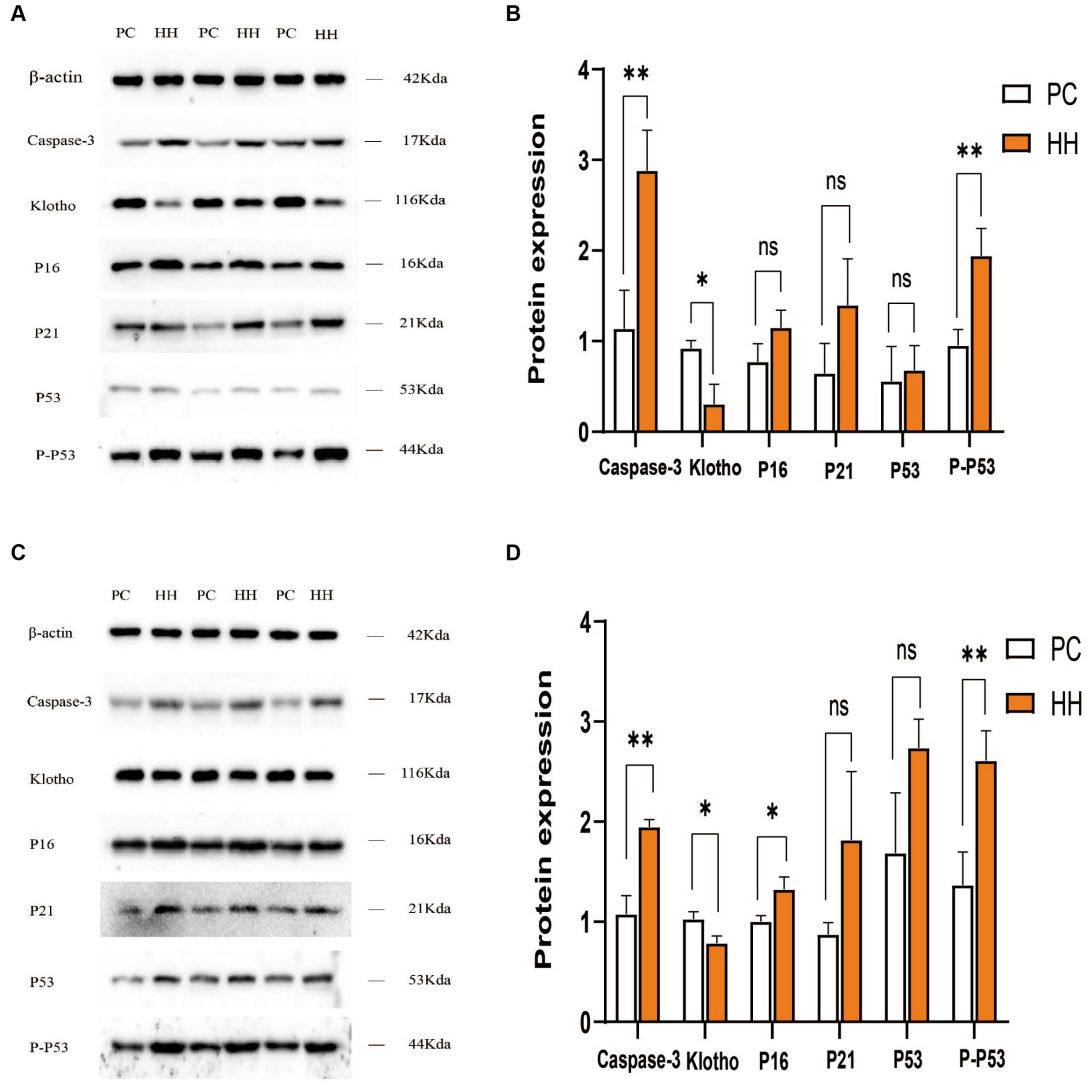
Comparison of expression of related proteins in the hippocampus and frontal cortex of mice in the two groups. **(A,B)** Western blotting and statistical maps of each protein in the hippocampus. **(C,D)** Western blotting and statistical maps of each protein in frontal cortex tissue *p < 0.05; **p <0.01.

Compared with PC group, the expression of caspase3 and P-P53 protein in the hippocampus of the HH group was significantly increased (*p* < 0.01), while klotho protein expression was decreased (*p* < 0.05). There were no significant changes in the expression of P16, P21, and P53 (*p* > 0.05; Figures 3A,B). Compared with the PC group, the expression of caspase3, P16, and P-P53 in the cortex of mice in the HH group was significantly increased (*p* < 0.01, *p* < 0.05, and *p* < 0.01, respectively), while klotho protein expression decreased (*p* < 0.05). There were no significant changes in the expression of P21 and P53 (*p* > 0.05; Figures 3C,D).

### Brain GM volume changes

**Figure 4:**
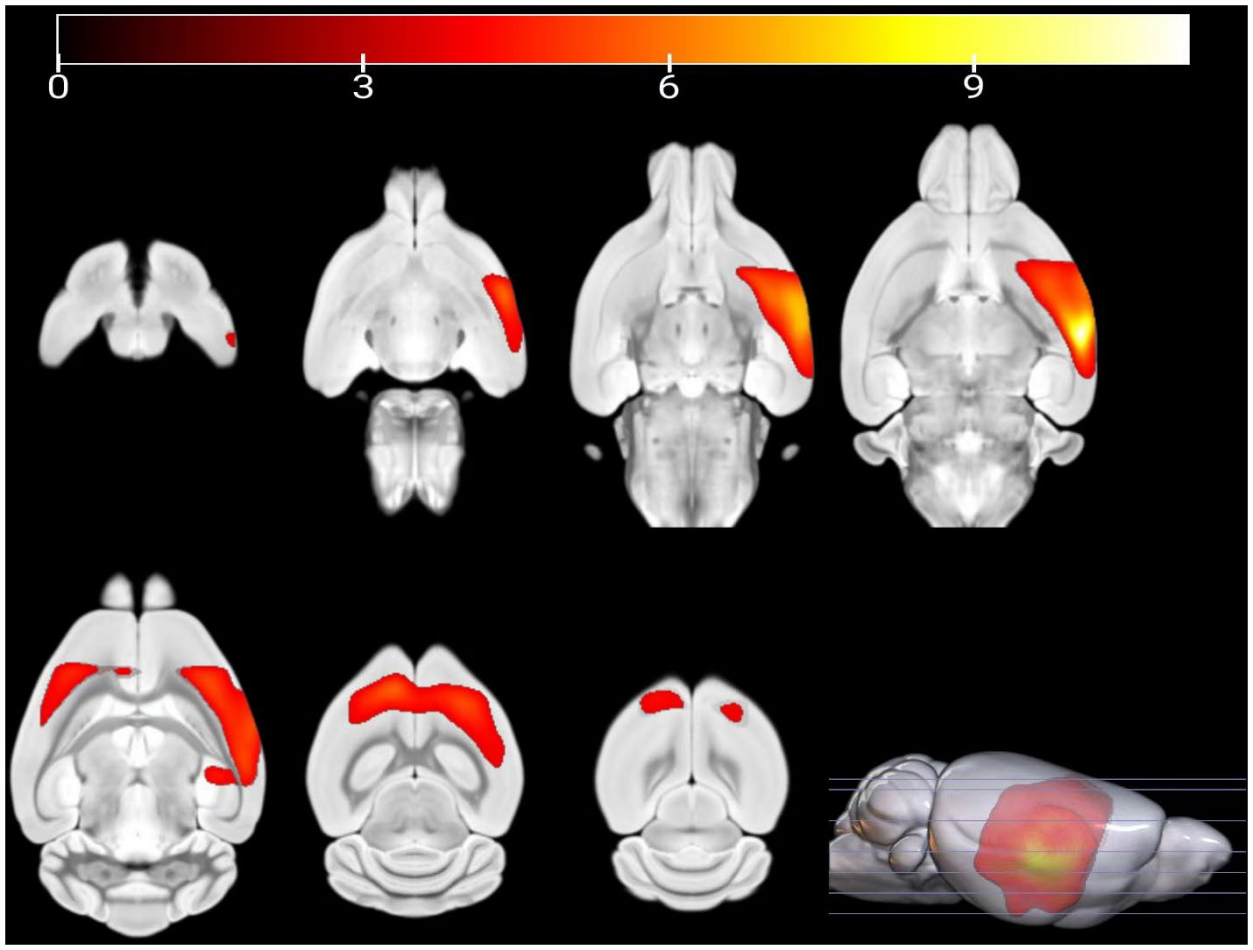
Statistical parameters of GM volume difference between the PC and HH groups. GM volumes in the left visceral area, left caudate nucleus, and left piriform cortex were decreased in the HH group (voxel level *p* < 0.001, cluster level *p* < 0.05 FWE correction). The color bar indicates the *t*-value.

Compared with PC group, the GM volume of the left visceral region, left caudate nucleus, and left piriform cortex region were decreased in the HH group (Table 2 and Figure 4).

**Table 2:** Montreal Neurological Institute (MNI) coordinates and relevant statistical data of cerebellar regions with reduced GM in the HH and PC groups.

ROIs name	Peak MNI coordinate			T value	Cluster size
	X	Y	Z		
Visceral_area/_layer_5_Left, Caudoputamen_Left, Piriform_area_Left	39	−5.7	−2.4	11.10	114,158

### ALFF value changes

**Figure 5:**
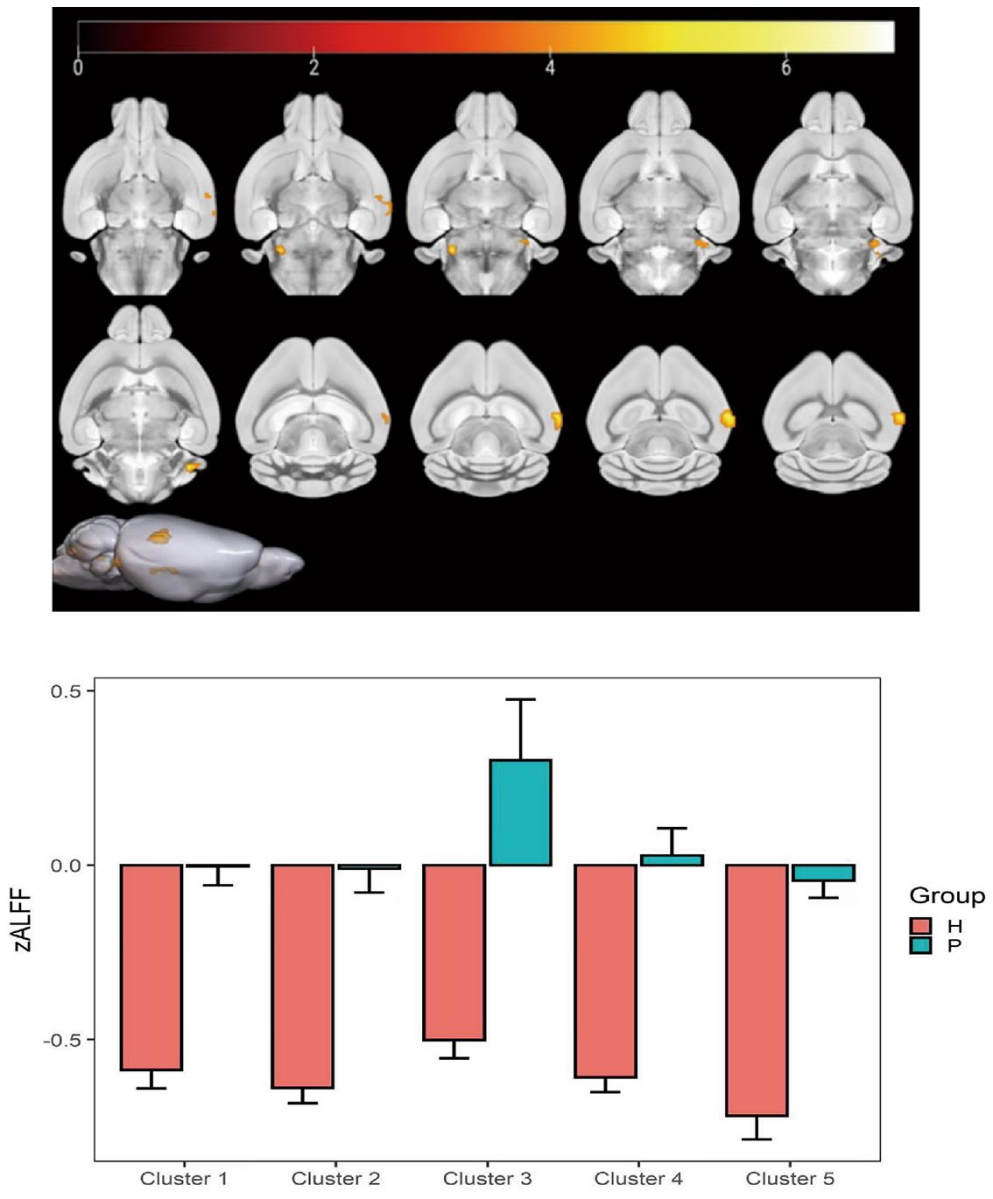
Statistical parameters comparing ALFF values between the PC and HH groups. In the HH group, the ALFF of the left ungranular posterior insula, right reticular nucleus of small cells, left flocs, left paratropon, and left primary auditory area were decreased (voxel level *p* < 0.001, cluster size >10, uncorrected). The color bar indicates the *t*-value.

**Figure 6:**
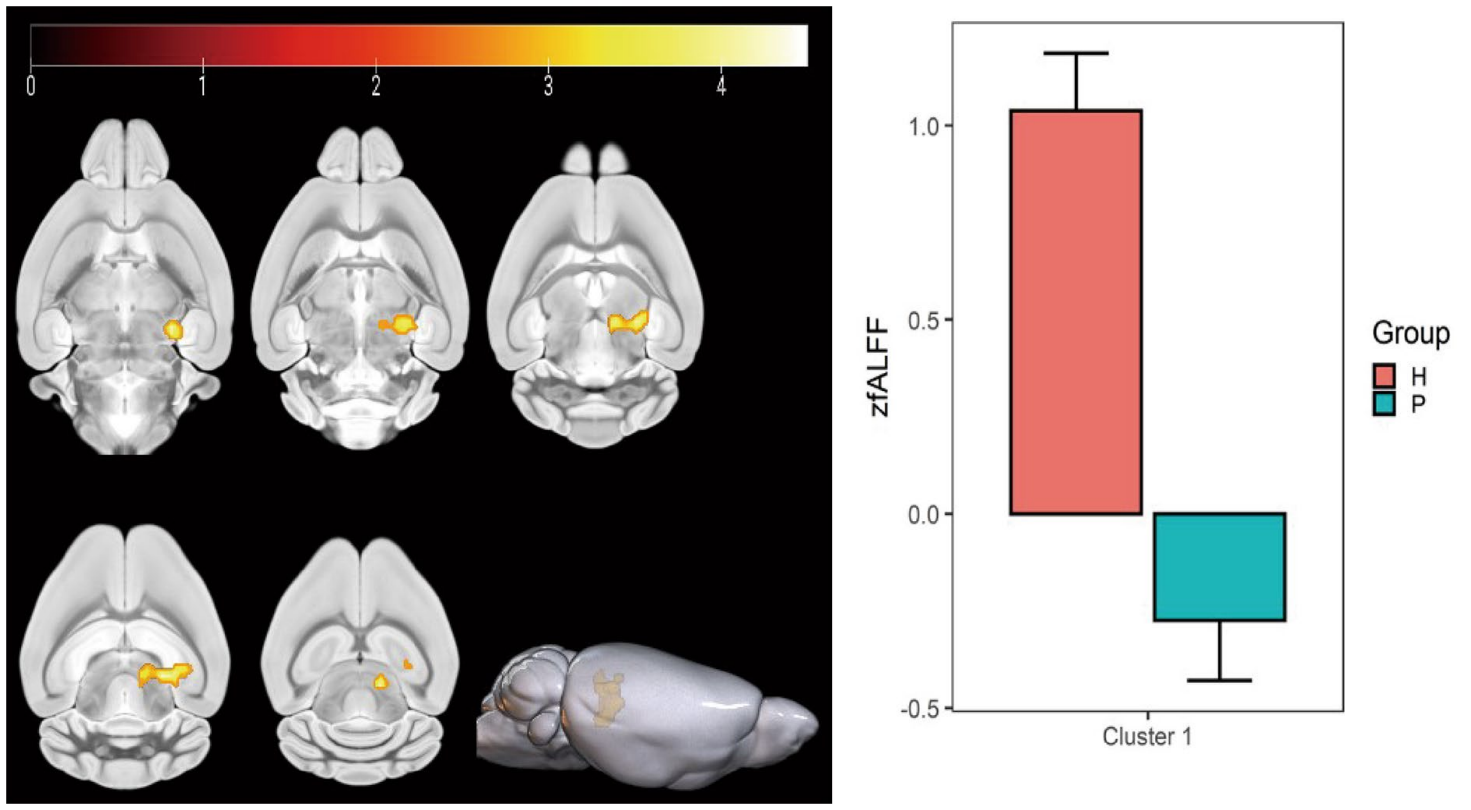
Statistical parameters comparing fALFF values between the PC and HH groups. fALFF in the left upper colliculus GM layer increased in the HH group (voxel level *p* < 0.001, cluster level *p* < 0.05, FWE corrected, cluster size = 199). The color bar indicates the *t*-value.

Compared with the PC group, the ALFF of the left ungranular posterior insula, right reticular nucleus of small cells, left flocs, left paratropon and left primary auditory area in the HH group were decreased (Table 3 and Figure 5). Compared with PC group, the HH group showed increased fALFF in the left superior colliculus GM layer (Table 4 and Figure 6).

**Table 3:** MNI coordinates and relevant statistical data of ALFF reduction between the PC and HH groups.

ROIs name	Peak MNI coordinate			*T* value	Cluster size
	X	Y	Z		
Left ungranular posterior insula	37.2	−7.0929	−6.8	5.05	15
Right reticular nucleus	−16.8	−41.0929	−6.8	7.14	13
Left flocs	25.2	−37.0929	−2.8	4.97	17
Left paratropon	27.2	−45.0929	1.2	6.52	11
Left primary auditory area	39.2	−11.0929	15.2	6.22	55

**Table 4:** MNI coordinates and relevant statistical data of fALFF increase in the PC and HH groups.

ROIs name	Peak MNI coordinate			*T* value	Cluster size
	X	Y	Z		
Left superior colliculus GM layer	15.2	−19.0929	11.2	4.96	199

### ReHo value changes

**Figure 7:**
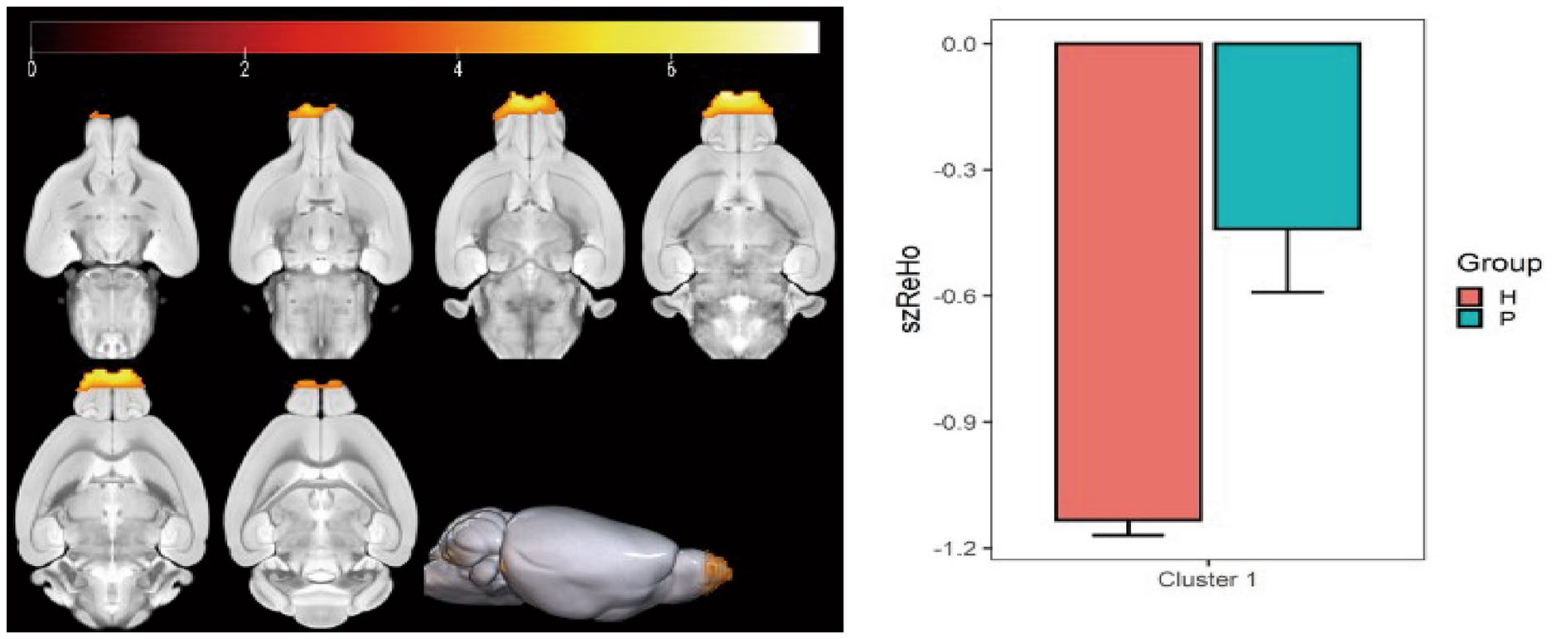
Statistical parameters of ReHo differences between the PC and HH groups. In the HH group, the ReHo in the left and right olfactory regions decreased (voxel level *p* < 0.001, cluster level *p* < 0.05, FWE corrected, cluster size = 499). The color bar indicates the *t*-value.

**Figure 8:**
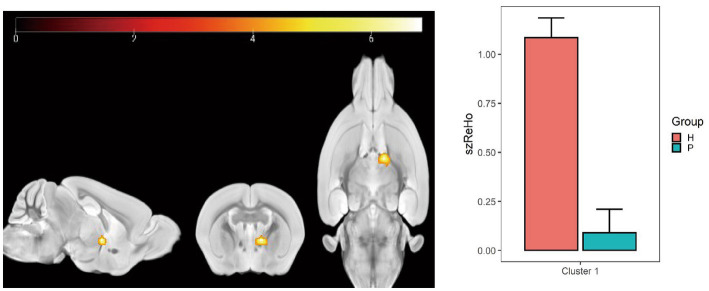
Statistical parameters of ReHo difference between the PC and HH groups. In the HH group, the left BNST ReHo was increased (voxel level *p* < 0.001, cluster size >10, uncorrected). The color bar indicates the *t*-value.

Compared with the PC group, the ReHo value in the left and right olfactory regions was decreased in the HH group (Table 5 and Figure 7), while the ReHo value in the left bed nucleus of the striatus was increased (Table 6 and Figure 8).

**Table 5:** MNI coordinates and statistical data of ReHo differences between the PC and HH groups with supporting statistical data.

ROIs name	Peak MNI coordinate			*T* value	Cluster size
	X	Y	Z		
Left and right olfactory regions	−4.8	66.9071	−4.8	7.39	499

**Table 6:** MNI coordinates and statistical data of ReHo elevation in PC and HH groups.

ROIs name	Peak MNI coordinate			*T* value	Cluster size
	X	Y	Z		
Left bed nucleus of the striatus	9.2	4.9071	−6.8	7.41	26

### FC changes

**Figure 9:**
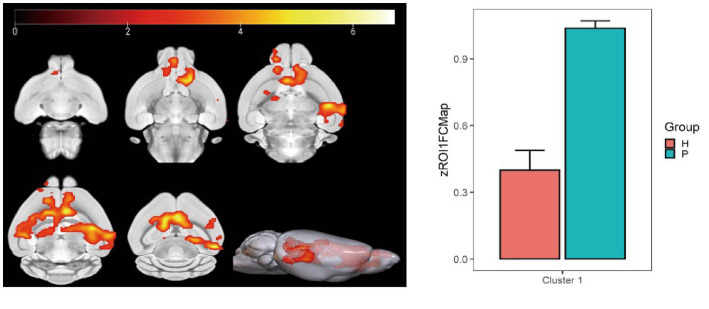
Statistical parameters of FC difference between the PC and HH groups using the caudate nucleus as the seed point. FC decreased in the left and right caudate nucleus, right principal olfactory bulb, left hippocampal CA3 area, and right corpus callosum in the HH group (voxel level *p* < 0.001, cluster level *p* < 0.05, FWE correction, cluster size = 3,951). The color bar indicates the *t*-value.

**Figure 10:**
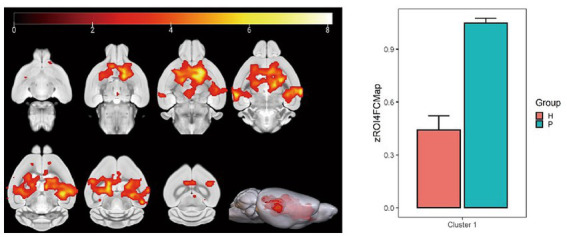
Statistical parameters of FC difference between the PC and HH groups using the cingulate gyrus as the seed point. FC in the left and right caudate nucleus, left and right hippocampal CA3 region, and left hippocampal CA1 region decreased in the HH group (voxel level *p* < 0.001, cluster level *p* < 0.05, FWE corrected, cluster size = 5,489). The color bar indicates the *t*-value.

Using the caudate nucleus as the seed point, whole-brain correlation analysis showed decreased FC with the left and right caudate nucleus, right principal olfactory bulb, left hippocampal CA3 area, and right corpus callosum in the HH group compared with the PC group (Table 7 and Figure 9). Using the cingulate gyrus as the seed point, FC with the left and right caudate nucleus, left and right hippocampal CA3 region, and left hippocampal CA1 region decreased in the HH group compared with the PC group (Table 8 and Figure 10).

**Table 7:** MNI coordinates and statistical data of FC reduction in the HH group using the caudate nucleus as the seed point.

ROIs name	Peak MNI coordinate			*T* value	Cluster size
	X	Y	Z		
Left and right caudate nucleus, right principal olfactory bulb, left hippocampal CA3 area, and right corpus callosum	1.2	22.9071	−4.8	6.94	3,951

**Table 8:** MNI coordinates and statistical of FC reduction in the HH group using the cingulate gyri as the seed point.

ROIs name	Peak MNI coordinate			*T* value	Cluster size
	X	Y	Z		
Left and right caudate nucleus, left and right hippocampal CA3 region, and left hippocampal CA1 region	45.2	−9.0929	3.2	8.79	5,489

**Figure 11:**
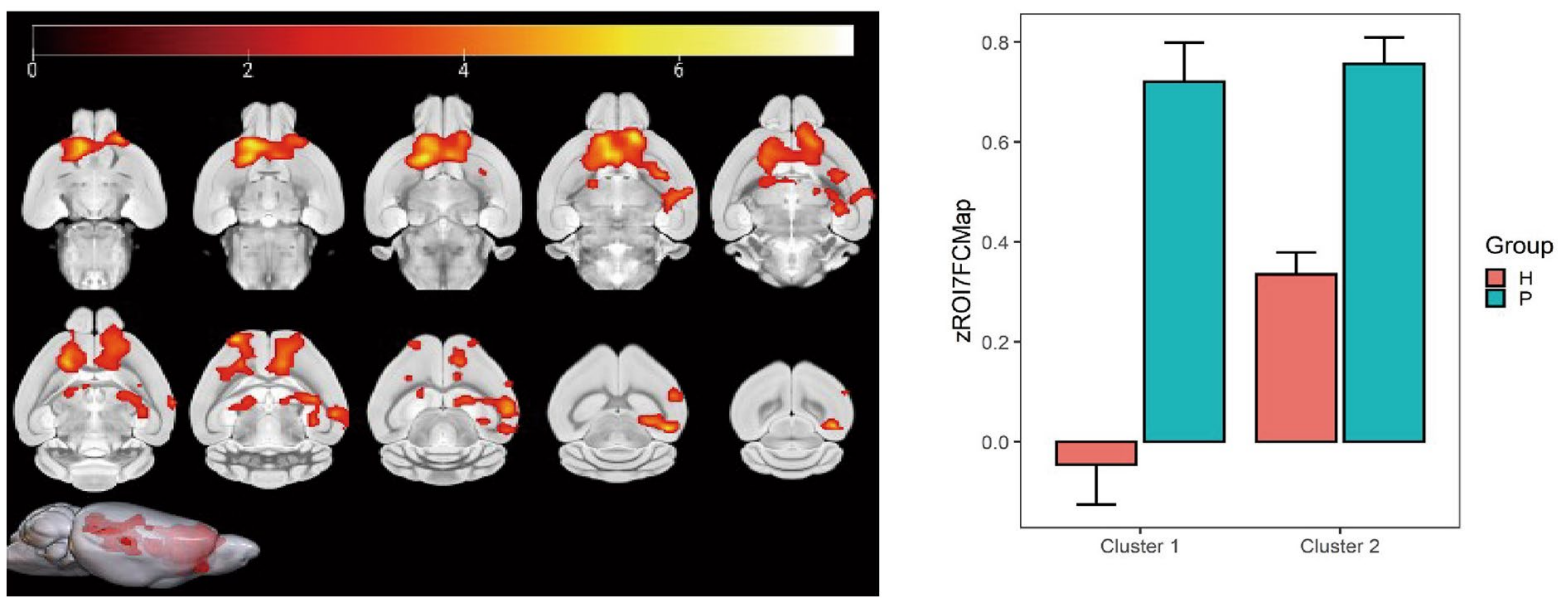
Statistical data of FC differences between the PC and HH groups using the globus pallidus as the seed point. In the HH group, FC in the left and right caudate nucleus, left and right anterior olfactory nucleus, and right olfactory region decreased (voxel level *p* < 0.001, cluster level *p* < 0.05, FWE corrected, cluster size = 2,619). FC also decreased in the left hippocampal CA3 region (voxel level *p* < 0.001, cluster level *p* < 0.05, FWE corrected, 5 cluster size = 776). The color bar indicates the *t*-value.

Using the globus pallidus as the seed point, correlation analysis using whole-brain voxels showed that FC in left and right caudate nucleus, left and right anterior olfactory nucleus, right olfactory region, and left hippocampus CA3 region decreased in the HH group compared with the PC group (Table 9 and Figure 11).

**Table 9:** MNI coordinates and related statistical data of FC differences between the PC and HH groups using the pallidum as the seed point.

ROIs name	Peak MNI coordinate			*T* value	Cluster size
	X	Y	Z		
Left and right caudate nucleus, left and right anterior olfactory nucleus, right olfactory region	−4.8	22.9071	−4.8	6.67	2,619
Left hippocampus CA3 region	37.2	−13.0929	11.2	8.04	776

**Figure 12:**
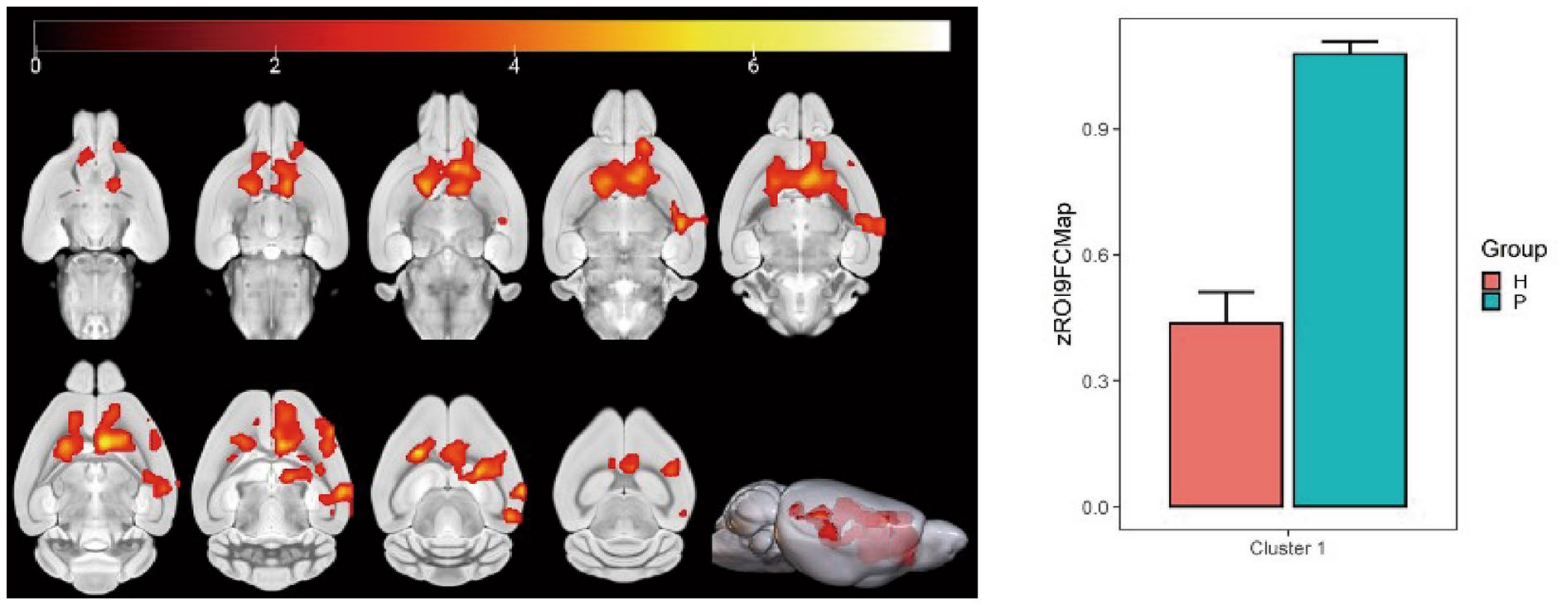
Statistical parameters of FC difference between the PC and HH groups using the hippocampus as the seed point. FC decreased in the left and right caudate nucleus, left corpus callosum, left anterior olfactory nucleus, and left lateral septal nucleus in the HH group (voxel level *p* < 0.001, cluster level *p* < 0.05, FWE corrected, cluster size = 2,832). The color bar indicates the *t*-value.

Using the hippocampus as the seed point, correlation analysis with whole-brain voxels showed that FC decreased in the left and right caudate nucleus, left corpus callosum, left anterior olfactory nucleus, and left lateral septal nucleus in the HH group compared with the PC group (Table 10 and Figure 12).

**Table 10:** MNI coordinates and related statistical data of FC differences between the PC and HH groups using the hippocampus as the seed point.

ROIs name	Peak MNI coordinate			*T* value	Cluster size
	X	Y	Z		
Left and right caudate nucleus, left corpus callosum, left anterior olfactory nucleus, and left lateral septal nucleus	31.2	−7.0929	−4.8	7.82	2,832

### Fractional anisotropy (FA) changes in the hippocampus

**Figure 13:**
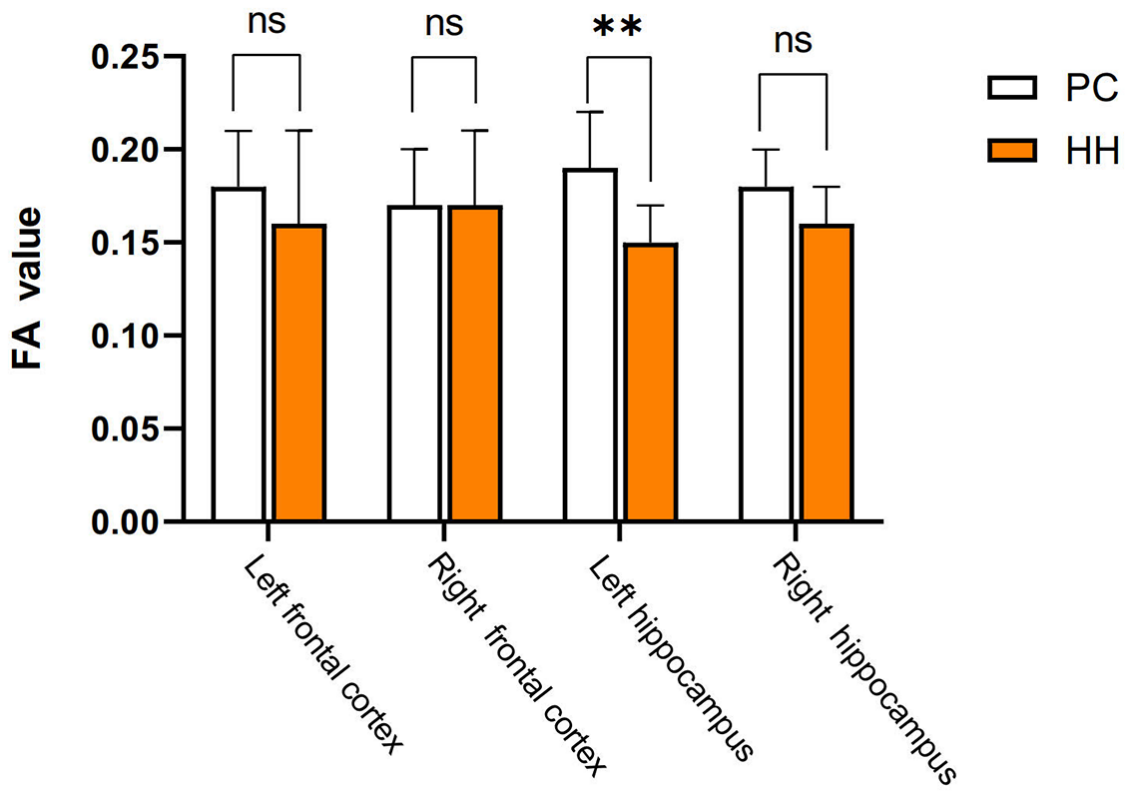
Comparison of cortical and hippocampal FA values between the PC and HH groups **p <0.01.

Compared with the PC group, the left hippocampal FA value was significantly decreased in the HH group (*p* < 0.05; Table 11 and Figure 13).

**Table 11:** Comparison of FA values between the PC and HH groups (mean ± SD).

Group	*N*	Cortex		Hippocampus	
		L	R	L	R
PC	8	0.18 ± 0.03	0.17 ± 0.03	0.19 ± 0.03	0.18 ± 0.02
HH	8	0.16 ± 0.05	0.17 ± 0.04	0.15 ± 0.02	0.16 ± 0.02
*t*-value		0.591	−0.098	3.371	1.484
*p*-value		0.564	0.924	0.005	0.160

### Cerebral blood flow (CBF) changes in the hippocampus

**Figure 14:**
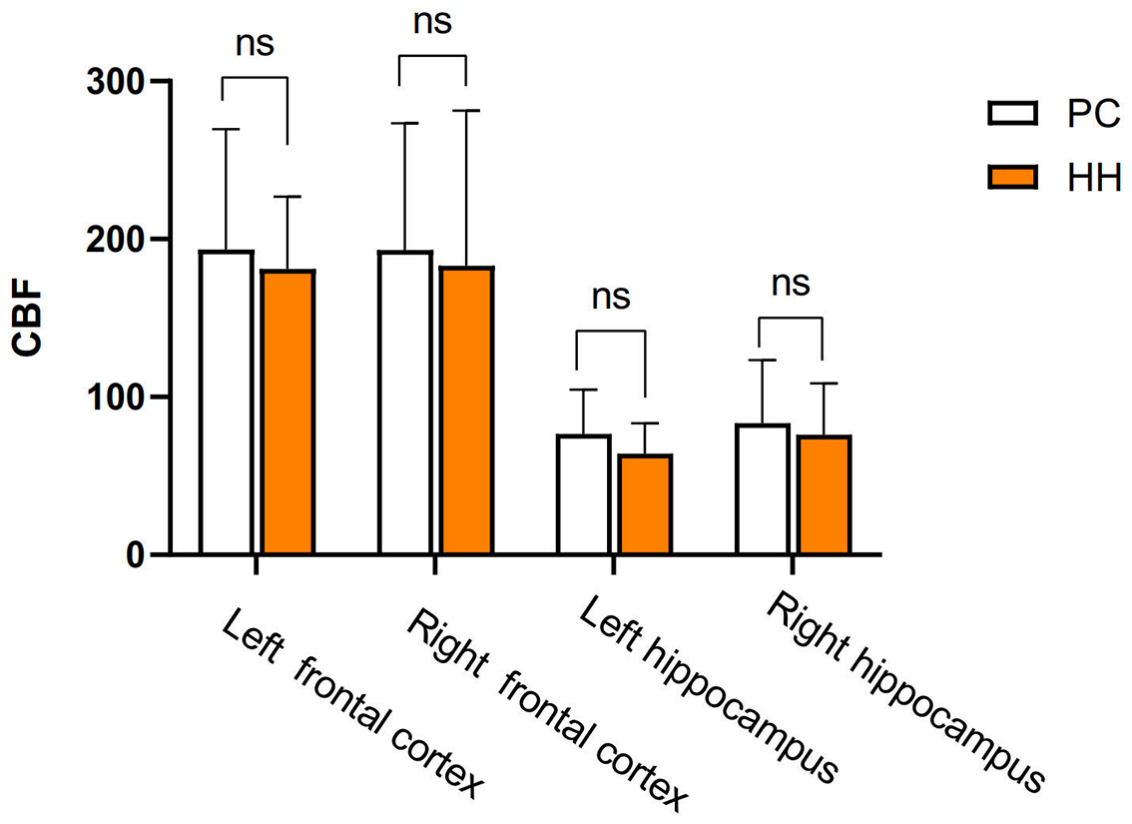
Comparison of cortical and hippocampal CBF between the two groups.

There were no significant differences in the CBF values of the bilateral cortex and hippocampus between the PC and HH groups (*p* > 0.05; Table 12 and Figure 14).

**Table 12:** Comparison of CBF ml/(100 g. min) values between the PC and HH groups (mean ± SD).

Group	*N*	Cortex		Hippocampus	
		L	R	L	R
PC	8	193.49 ± 76.02	193.01 ± 80.25	76.53 ± 28.11	83.43 ± 39.83
HH	6	181.03 ± 45.96	183.16 ± 98.10	64.21 ± 19.27	76.13 ± 32.55
*t*-value		0.354	0.207	0.919	0.366
*p*-value		0.730	0.840	0.376	0.721

### Carotid internal diameter changes

**Figure 15:**
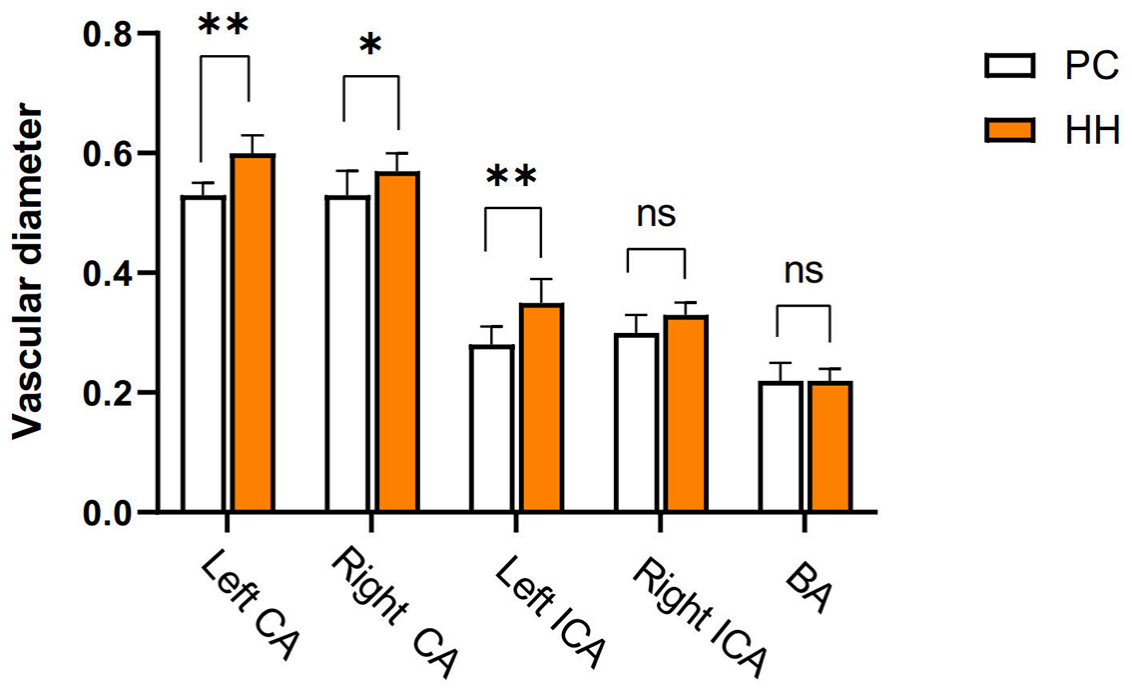
Comparison of blood vessel diameter between the PC and HH groups *p < 0.05; **p <0.01.

Compared with the PC group, the internal diameters of the bilateral common carotid artery and left internal carotid artery were significantly increased in the HH group (*p* < 0.05; Table 13 and Figure 15).

**Table 13:** Comparison of vascular inner diameter (mm) between the PC and HH groups (mean ± SD).

Group	*N*	CCA		ICA		BA
		L	R	L	R	
PC	8	0.53 ± 0.02	0.53 ± 0.04	0.28 ± 0.03	0.30 ± 0.03	0.22 ± 0.03
HH	8	0.60 ± 0.03	0.57 ± 0.03	0.35 ± 0.04	0.33 ± 0.02	0.22 ± 0.02
*t*-value		−4.414	−2.167	−3.455	−2.080	0.000
*p*-value		0.001	0.048	0.004	0.057	1.000

CCA, common carotid artery; ICA, internal carotid artery; BA, basilar artery.

## Discussion

At present, the effects of chronic hypoxia exposure on brain aging-related proteins are unknown because multi-modal imaging studies have not been performed. This study was the first to use a mouse model of chronic hypoxia exposure in a high-altitude hypoxia environment to reveal the effects of chronic hypoxia exposure at high altitude on brain aging related proteins and related imaging characteristics.

As a morphological index of nerve cell functional activity, Nishi bodies can indirectly reflect the degree of neuronal injury. In the present study, it was found that neurons in the hippocampal CA1 region and frontal cortex of mice significantly decreased after 24 weeks of exposure to a low-oxygen environment at high altitude. These results indicate that long-term hypoxia stimulation can damage neurons, which is consistent with previous research findings (Ji et al., 2021a,b).

Electron microscopy revealed that most mitochondria and endoplasmic reticulum of neurons in the HH group showed morphological changes. We believe that neuronal damage is the direct cause of the decline in brain function caused by a low oxygen environment, and we speculate this is also an early manifestation of the brain aging process. Related studies have demonstrated increased neurodegeneration and mitochondrial morphological changes in the hippocampal CA3 region of young rats exposed to chronic hypoxia, suggesting neuronal aging (Biswal et al., 2016).

Caspase-3 is considered a key regulator of nerve cell apoptosis (Means et al., 2016) and is involved in brain aging (Gionchiglia et al., 2021). In this study, expression of Caspase-3 in the hippocampus and frontal cortex of mice in the HH group was significantly increased at both the gene level and protein level. The klotho gene is recognized as an anti-aging gene and its overexpression can prolong the lifespan of mice (Zhu et al., 2019). The absence of the klotho gene can lead to premature aging (Ullah and Sun, 2019). This gene is crucial in regulating aging in mammals and the development of age-related diseases, and defective expression of the Klotho gene can cause aging of almost all organs and systems in mice (Anamizu et al., 2005). In the present study, expression of klotho in the hippocampus and frontal cortex of mice in the HH group was significantly decreased. These findings suggest that long-term high altitude exposure can affect expression of the klotho gene and protein in mouse brain tissue, which may weaken the anti-aging effect of klotho protein. Expression of the P16 gene is one of the most powerful indicators of cellular senescence and increases exponentially with age (Smitherman et al., 2020). Overexpression of P16 can accelerate cell senescence, resulting in body damage and accelerated senescence (Shen et al., 2020). When the P16 gene is deficient DNA repair ability is enhanced, leading to deceleration of telomere shortening and reduced cellular senescence. P16 is closely related to the P21 and P53 genes and is involved in regulation of the cell cycle (Terzi et al., 2016). Studies have shown that the mRNA levels of P16 and P21 are increased in tissues of premature aging mice and elderly mice (Yousefzadeh et al., 2020). In addition to inducing cell growth arrest and apoptosis, the activation of P53 gene also regulates cell senescence and tissue senescence (Rufini et al., 2013). There is some evidence that increased activity of the P53 gene promotes aging in the body (Rodier et al., 2007). In this study, expression of the P16, P21, and P53 genes was up-regulated in the HH group, and the expression of P16, P21, P53, and P-P53 proteins in the hippocampus and frontal cortex tissues of HH mice was also enhanced. These results suggest that exposure to a high altitude, low oxygen environment may interfere with the cell regulation cycle and lead to accelerated aging of neurons.

MRI has been previously applied to study changes in brain structure and function in hypoxic environments. However, few studies have used multimodal MRI to comprehensively evaluate brain structural and functional changes after exposure to high altitude. VBM provides a quantitative and comprehensive assessment of brain anatomical changes (Ashburner and Friston, 2000) and has been widely used to study brain morphological changes caused by various diseases (Brenneis et al., 2003; Shigemoto et al., 2013). Variation in GM density between the HH and PC groups was analyzed by the VBM technique. The results showed that the GM volume of multiple brain regions of mice exposed to a high altitude environment decreased significantly. A decreased GM density may relate to increased release of brain metabolites and glutamate from nerve cells after hypoxia (Zatorre et al., 2012). In this study, decreased GM density was observed in the left caudate nucleus, visceral area, and piriform cortex, as well as other areas. Notably, the caudate nucleus is related to emotional regulation and visual memory performance (Bartrés-Faz et al., 2002; Kious et al., 2019). The piriform cortex is the largest component of the olfactory cortex and, like the hippocampus, belongs to the limbic system. In addition to participating in olfaction (Bekkers and Suzuki, 2013), this region is closely related to learning, memory, and sleep (Barnes and Wilson, 2014; Reuveni et al., 2018). Thus, it can be speculated that decreased GM density in these regions may relate to memory loss, depression, and sleep disturbance after long-term exposure to high altitude hypoxia.

The results also showed that ALFF decreased in the left ungranular posterior insula, right reticular nucleus of small cells, left flocculus, left paratropium, and left primary auditory area, while fALFF increased in the left superior colliculus GM layer, in the HH group. Furthermore, ReHo in the left and right olfactory areas decreased, while ReHo in the left bed nucleus increased. ALFF is an important parameter that reflects the intensity of spontaneous neural activity in the resting state (Zang et al., 2007) and can show spontaneous firing activity of neurons in different brain functional areas. ReHo mainly reflects the consistency of local neuronal activity of adjacent voxels. Reduced ReHo values indicate a disturbance of neuronal activity and time series in local brain functional areas, which may indicate severe brain dysfunction (Chen et al., 2019). Our results suggest that the spontaneous activity of neurons in multiple brain regions is altered after chronic hypoxic exposure and that neuronal activity is disordered. This is a potential cause of decreased brain function and accelerated brain aging after exposure to a low oxygen environment at high altitude. In this study, nine regions of interest were selected based on ALFF and ReHo results and previous studies. A whole-brain analysis method was then applied to study FC changes between brain regions after exposure to high altitude and low oxygen in mice. We found that FC between the bilateral caudate nucleus and thalamus, corpus callosum, cingulate gyrus, globus pallidus, and hippocampus was weakened after high altitude exposure. These regions play important roles in sensory processing, executive function, memory, and other important cognitive functions. A previous study reported that the strength of the connection between the caudate nucleus and the hippocampus is correlated with memory performance (Müller et al., 2018). The hippocampus, entorhinal cortex, and cingulate gyrus are all important components of the limbic system and have extensive connections with other brain functional areas such as the thalamus, brain stem and neocortex (Bubb et al., 2017), playing an important role in emotional experience, emotional expression, and episodic memory formation. Numerous studies (Kious et al., 2018; Cao et al., 2021; Wang et al., 2021) have reported a higher prevalence of depression in people exposed to low-oxygen environment at high altitudes compared to people in plains areas, as well as significant decreases in cognitive function (Davis et al., 2015; Das et al., 2018). The finding of weakened FC among multiple brain regions in this study indicates that connections between the caudate nucleus, hippocampus, thalamus, and other regions are affected by chronic hypoxia. Thus, we speculate that the decline in cognitive function and impaired emotional expression after exposure to high altitude hypoxia may relate to changes in FC.

By calibrating the movement direction of water molecules in tissues, DTI can clearly visualize anisotropy characteristics of white matter fibers (Clark and Werring, 2002) and is currently the only imaging method that can noninvasively reveal fiber structures inside the brain (Huisman et al., 2004). As FA values indirectly indicate the integrity of white matter fiber bundles, it can be used as a measure of WM integrity (Gulani and Sundgren, 2006). A higher FA value indicates a closer arrangement of fiber bundles in white matter and a stronger directivity. Reduced FA values are commonly observed in inflammation, edema, glial cell proliferation, and demyelination (Hemanth Kumar et al., 2014). In the present study, we found that the FA value of the left hippocampus was significantly reduced in the HH group, suggesting that chronic hypoxic environmental exposure at high altitude may lead to changes in white matter of the brain, which is consistent with related studies (Hong et al., 2013).

This study used CBD values and measured the internal diameter of arteries by ASL and MRA imaging. We found no difference in the CBF value between the PC and HH groups, but the internal diameters of the bilateral common carotid artery and left internal carotid artery were significantly enlarged in the HH group. Brain tissue is extremely sensitive to changes in oxygen demand and partial pressure. In the case of acute hypobaric hypoxia, the body can ensure a supply of oxygen and energy to the brain by strengthening ventilation, accelerating the heart rate, increasing blood pressure, and changing the arterial blood gas balance. Acute hypoxia and hypoxia can stimulate the nervous and endocrine systems and may lead to the release of cytokines, such as endothelium-derived nitric oxide (NO), that cause systemic vasoconstriction, thereby increasing the CBF rate (Wu et al., 2016). With gradual adaptation of the body to a high altitude environment, cerebrovascular reactivity, and vasomotor function are gradually restored and CBF slowly decreases to a level close to the normal range (Lucas et al., 2011; Ainslie and Subudhi, 2014). Although there are differences in CBF among individuals, changes in CBF after moving from a plains environment to a high altitude are generally consistent with the above result (Hoiland et al., 2018). Therefore, we believe that both the bilateral common carotid artery and left internal carotid artery are significantly enlarged after chronic hypoxic exposure, which may be an adaptive structural change to adjust CBF stability.

Our study did not investigate the relationship between morphological changes in neurons and abnormal expression of related proteins and imaging changes. However, it can be speculated that accelerated apoptosis and increased necrosis of neurons caused by abnormal expression of related proteins after exposure to chronic hypoxia at high altitude is the basis for changes in brain structure and function, and may also cause accelerated aging of the brain after exposure to hypoxia at a high altitude. However, the underlying mechanisms warrant further study in the future.

## Data availability statement

The raw data supporting the conclusions of this article will be made available by the authors, without undue reservation.

## Ethics statement

The animal study was approved by Research Ethics Committee of Qinghai Provincial People’s Hospital. The study was conducted in accordance with the local legislation and institutional requirements.

## Author contributions

YC: Data curation, Writing – original draft. SC: Formal Analysis, Investigation, Writing – original draft. R-LG: Supervision, Validation, Writing – review & editing. HB: Project administration, Software, Visualization, Writing – review & editing. YM: Methodology, Software, Visualization, Writing – original draft. WJ: Writing – review & editing.

## Footnotes

^1^ http://www.fil.ion.ucl.ac.uk/spm

^2^ http://www.rfmri.org/dpabi

## References

[ref1] Ainslie P. N. Subudhi A. W. (2014). Cerebral blood flow at high altitude. High Alt. Med. Biol. 15, 133–140. doi: 10.1089/ham.2013.1138 24971767

[ref2] Anamizu Y. Kawaguchi H. Seichi A. Yamaguchi S. Kawakami E. Kanda N.. (2005). Klotho insufficiency causes decrease of ribosomal RNA gene transcription activity, cytoplasmic RNA and rough ER in the spinal anterior horn cells. Acta Neuropathol. 109, 457–466. doi: 10.1007/s00401-004-0971-7, PMID: 15834732

[ref3] Ashburner J. Friston K. J. (2000). Voxel-based morphometry--the methods. Neuroimage 11, 805–821. doi: 10.1006/nimg.2000.0582, PMID: 10860804

[ref4] Bao H. He X. Wang F. Kang D. (2022). Study of brain structure and function in chronic mountain sickness based on fMRI. Front. Neurol. 12:763835. doi: 10.3389/fneur.2021.763835 35069409 PMC8777079

[ref5] Barnes D. C. Wilson D. A. (2014). Sleep and olfactory cortical plasticity. Front. Behav. Neurosci. 8:134. doi: 10.3389/fnbeh.2014.00134 24795585 PMC4001050

[ref6] Bartrés-Faz D. Junqué C. Serra-Grabulosa J. M. López-Alomar A. Moya A. Bargalló N.. (2002). Dopamine DRD2 Taq I polymorphism associates with caudate nucleus volume and cognitive performance in memory impaired subjects. Neuroreport 13, 1121–1125. doi: 10.1097/00001756-200207020-00010, PMID: 12151753

[ref7] Bekkers J. M. Suzuki N. (2013). Neurons and circuits for odor processing in the piriform cortex. Trends Neurosci. 36, 429–438. doi: 10.1016/j.tins.2013.04.005 23648377

[ref8] Biswal S. Sharma D. Kumar K. Nag T. C. Barhwal K. Hota S. K.. (2016). Global hypoxia induced impairment in learning and spatial memory is associated with precocious hippocampal aging. Neurobiol. Learn. Mem. 133, 157–170. doi: 10.1016/j.nlm.2016.05.011, PMID: 27246251

[ref9] Brenneis C. Seppi K. Schocke M. F. Müller J. Luginger E. Bösch S.. (2003). Voxel-based morphometry detects cortical atrophy in the Parkinson variant of multiple system atrophy. Mov. Disord. 18, 1132–1138. doi: 10.1002/mds.10502, PMID: 14534916

[ref10] Bubb E. J. Kinnavane L. Aggleton J. P. (2017). Hippocampal - diencephalic - cingulate networks for memory and emotion: an anatomical guide. Brain Neurosci. Adv. 1:2398212817723443. doi: 10.1177/2398212817723443 28944298 PMC5608081

[ref11] Cao Y. Li G. Xue J. Zhang G. Gao S. Huang Y.. (2021). Depression and related factors in patients with Parkinson’s disease at high altitude. Neuropsychiatr. Dis. Treat. 17:1353. doi: 10.2147/NDT.S300596 33986595 PMC8110268

[ref12] Chen X. Li H. Zhang Q. Wang J. Zhang W. Liu J.. (2019). Combined fractional anisotropy and subcortical volumetric abnormalities in healthy immigrants to high altitude: a longitudinal study. Hum. Brain Mapp. 40, 4202–4212. doi: 10.1002/hbm.24696 31206892 PMC6865614

[ref14] Clark C. A. Werring D. J. (2002). Diffusion tensor imaging in spinal cord: methods and applications - a review. NMR Biomed. 15, 578–586. doi: 10.1002/nbm.788, PMID: 12489104

[ref15] Das S. K. Dhar P. Sharma V. K. Barhwal K. Hota S. K. Norboo T.. (2018). High altitude with monotonous environment has significant impact on mood and cognitive performance of acclimatized lowlanders: possible role of altered serum BDNF and plasma homocysteine level. J. Affect. Disord. 237, 94–103. doi: 10.1016/j.jad.2018.04.106 29803101

[ref16] Davis J. E. Wagner D. R. Garvin N. Moilanen D. Thorington J. Schall C. (2015). Cognitive and psychomotor responses to high-altitude exposure in sea level and high-altitude residents of Ecuador. J. Physiol. Anthropol. 34:2. doi: 10.1186/s40101-014-0039-x 25649647 PMC4320830

[ref17] Gionchiglia N. Granato A. Merighi A. Lossi L. (2021). Association of Caspase 3 activation and H2AX γ phosphorylation in the aging brain: studies on untreated and irradiated mice. Biomedicine 9:1166. doi: 10.3390/biomedicines9091166 PMC8468010 34572352

[ref18] Gulani V. Sundgren P. C. (2006). Diffusion tensor magnetic resonance imaging. J. Neuroophthalmol. 26, 51–60. doi: 10.1097/01.wno.0000205978.86281.3e 16518169

[ref19] Hemanth Kumar B. S. Mishra S. K. Trivedi R. Singh S. Rana P. Khushu S. (2014). Demyelinating evidences in CMS rat model of depression: a DTI study at 7 T. Neuroscience 275:12. doi: 10.1016/j.neuroscience.2014.05.037 24881571

[ref20] Hoiland R. L. Howe C. A. Coombs G. B. Ainslie P. N. (2018). Ventilatory and cerebrovascular regulation and integration at high-altitude. Clin. Autonomic Res. 28, 423–435. doi: 10.1007/s10286-018-0522-2, PMID: 29574504

[ref21] Hong Y. J. Yoon B. Lim S. C. Shim Y. S. Kim J. Y. Ahn K. J.. (2013). Microstructural changes in the hippocampus and posterior cingulate in mild cognitive impairment and Alzheimer’s disease: a diffusion tensor imaging study. Neurol. Sci. 34, 1215–1221. doi: 10.1007/s10072-012-1225-4, PMID: 23109096

[ref22] Huisman T. A. Schwamm L. H. Schaefer P. W. Koroshetz W. J. Shetty-Alva N. Ozsunar Y.. (2004). Diffusion tensor imaging as potential biomarker of white matter injury in diffuse axonal injury. AJNR Am. J. Neuroradiol. 25, 370–376. PMID: 15037457 PMC8158566

[ref23] Ji W. Zhang Y. Ge R. L. Wan Y. Liu J. (2021a). NMDA receptor-mediated excitotoxicity is involved in neuronal apoptosis and cognitive impairment induced by chronic hypobaric hypoxia exposure at high altitude. High Alt. Med. Biol. 22, 45–57. doi: 10.1089/ham.2020.0127 33252277

[ref24] Ji W. Zhang Y. Luo J. Wan Y. Liu J. Ge R. L. (2021b). Memantine ameliorates cognitive impairment induced by exposure to chronic hypoxia environment at high altitude by inhibiting excitotoxicity. Life Sci. 270:119012. doi: 10.1016/j.lfs.2020.119012 33422543

[ref25] Kious B. M. Bakian A. Zhao J. Mickey B. Guille C. Renshaw P.. (2019). Altitude and risk of depression and anxiety: findings from the intern health study. Int. Rev. Psychiatry 31, 637–645. doi: 10.1080/09540261.2019.1586324, PMID: 31084447 PMC8530170

[ref26] Kious B. M. Kondo D. G. Renshaw P. F. (2018). Living high and feeling low: altitude, suicide, and depression. Harv. Rev. Psychiatry 26, 43–56. doi: 10.1097/HRP.0000000000000158, PMID: 29517615

[ref27] Lefferts W. K. DeBlois J. P. White C. N. Day T. A. Heffernan K. S. Brutsaert T. D. (2019). Changes in cognitive function and latent processes of decision-making during incremental ascent to high altitude. Physiol. Behav. 201:139. doi: 10.1016/j.physbeh.2019.01.002 30611763

[ref28] Lucas S. J. Burgess K. R. Thomas K. N. Donnelly J. Peebles K. C. Lucas R. A. I.. (2011). Alterations in cerebral blood flow and cerebrovascular reactivity during 14 days at 5050 m. J. Physiol. 589, 741–753. doi: 10.1113/jphysiol.2010.192534, PMID: 21041534 PMC3052440

[ref29] Maiti P. Singh S. B. Mallick B. Muthuraju S. Ilavazhagan G. (2008). High altitude memory impairment is due to neuronal apoptosis in hippocampus, cortex and striatum. J. Chem. Neuroanat. 36, 227–238. doi: 10.1016/j.jchemneu.2008.07.003, PMID: 18692566

[ref30] Manukhina E. B. Downey H. F. Shi X. Mallet R. T. (2016). Intermittent hypoxia training protects cerebrovascular function in Alzheimer’s disease. Exp. Biol. Med. 241, 1351–1363. doi: 10.1177/1535370216649060 PMC4950272 27190276

[ref31] Means J. C. Gerdes B. C. Kaja S. Sumien N. Payne A. J. Stark D. A.. (2016). Caspase-3-dependent proteolytic cleavage of tau causes neurofibrillary tangles and results in cognitive impairment during normal aging. Neurochem. Res. 41, 2278–2288. doi: 10.1007/s11064-016-1942-9 27220334 PMC4965284

[ref32] Müller N. C. J. Konrad B. N. Kohn N. Muñoz-López M. Czisch M. Fernández G.. (2018). Hippocampal-caudate nucleus interactions support exceptional memory performance. Brain Struct. Funct. 223, 1379–1389. doi: 10.1007/s00429-017-1556-2, PMID: 29138923 PMC5869896

[ref33] Reuveni I. Lin L. Barkai E. (2018). Complex-learning induced modifications in synaptic inhibition: mechanisms and functional significance. Neuroscience 381:105. doi: 10.1016/j.neuroscience.2018.04.023 29704609

[ref34] Rodier F. Campisi J. Bhaumik D. (2007). Two faces of p53: aging and tumor suppression. Nucleic Acids Res. 35, 7475–7484. doi: 10.1093/nar/gkm744 17942417 PMC2190721

[ref35] Rufini A. Tucci P. Celardo I. Melino G. (2013). Senescence and aging: the critical roles of p53. Oncogene 32, 5129–5143. doi: 10.1038/onc.2012.640 23416979

[ref36] Shen J. Song R. Fuemmeler B. McGuire K. P. Chow W. H. Zhao H. (2020). Biological aging marker p16 in T cells and breast Cancer risk. Cancers 12:3122. doi: 10.3390/cancers12113122, PMID: 33114473 PMC7692397

[ref37] Shigemoto Y. Matsuda H. Kamiya K. Maikusa N. Nakata Y. Ito K.. (2013). In vivo evaluation of gray and white matter volume loss in the parkinsonian variant of multiple system atrophy using SPM8 plus DARTEL for VBM. Neuroimage Clin. 2:491. doi: 10.1016/j.nicl.2013.03.017 24179801 PMC3777846

[ref38] Smitherman A. Wood W. Mitin N. Ayer Miller V. L. Deal A. M. Davis I. J.. (2020). Accelerated aging among childhood, adolescent, and young adult cancer survivors is evidenced by increased expression of p16 and frailty. Cancer 126, 4975–4983. doi: 10.1002/cncr.33112, PMID: 32830315 PMC7607511

[ref39] Terzi M. Y. Izmirli M. Gogebakan B. (2016). The cell fate: senescence or quiescence. Mol. Biol. Rep. 43, 1213–1220. doi: 10.1007/s11033-016-4065-0 27558094

[ref40] Ullah M. Sun Z. (2019). Klotho deficiency accelerates stem cells aging by impairing telomerase activity. J. Gerontol. A Biol. Sci. Med. Sci. 74, 1396–1407. doi: 10.1093/gerona/gly261 30452555 PMC6696722

[ref41] Wang F. Liu S. Zhang Q. Ng C. H. Cui X. Zhang D.. (2021). Prevalence of depression in older nursing home residents in high and low altitude regions: a comparative study. Front. Psych. 12. doi: 10.3389/fpsyt.2021.669234 PMC8257928 34239461

[ref42] Wu S. Hao G. Zhang S. Jiang D. Wuren T. Luo J. (2016). Cerebral vasoconstriction reactions and plasma levels of ETBR, ET-1, and eNOS in patients with chronic high altitude disease. Mol. Med. Rep. 14, 2497–2502. doi: 10.3892/mmr.2016.5555, PMID: 27485004 PMC4991730

[ref43] Xu G. Shi Y. K. Sun B. D. Liu L. E G. J. He S.. (2021). DL-3-n-butylphthalide im- improved physical and learning and memory performance of rodents exposed to acute and chronic hypobaric hypoxia. Mil. Med. Res. 8:23. doi: 10.1186/s40779-021-00314-7, PMID: 33766114 PMC7993509

[ref44] Yan X. Zhang J. Shi J. Gong Q. Weng X. (2010). Cerebral and functional adaptation with chronic hypoxia exposure: a multi-modal MRI study. Brain Res. 1348:21. doi: 10.1016/j.brainres.2010.06.024 20599837

[ref45] Yeo E. J. (2019). Hypoxia and aging. Exp. Mol. Med. 51, 1–15. doi: 10.1038/s12276-019-0233-3 PMC6586788 31221957

[ref46] Yousefzadeh M. J. Zhao J. Bukata C. Wade E. A. McGowan S. J. Angelini L. A.. (2020). Tissue specificity of senescent cell accumulation during physiologic and accelerated aging of mice. Aging Cell 19:e13094. doi: 10.1111/acel.13094 31981461 PMC7059165

[ref47] Zang Y. F. He Y. Zhu C. Z. Cao Q. J. Sui M. Q. Liang M.. (2007). Altered baseline brain activity in children with ADHD revealed by resting-state functional MRI. Brain Dev. 29, 83–91. doi: 10.1016/j.braindev.2006.07.002, PMID: 16919409

[ref48] Zatorre R. J. Fields R. D. Johansen-Berg H. (2012). Plasticity in gray and white: neuroimaging changes in brain structure during learning. Nat. Neurosci. 15, 528–536. doi: 10.1038/nn.3045, PMID: 22426254 PMC3660656

[ref49] Zhang G. Zhou S. M. Yuan C. Tian H. J. Li P. Gao Y. Q. (2013). The effects of short-term and long-term exposure to a high altitude hypoxic environment on neurobehavioral function. High Alt. Med. Biol. 14, 338–341. doi: 10.1089/ham.2012.1091, PMID: 24377340

[ref50] Zhu Z. Xia W. Cui Y. Zeng F. Li Y. Yang Z.. (2019). Klotho gene polymorphisms are associated with healthy aging and longevity: evidence from a meta-analysis. Mech. Ageing Dev. 178, 33–40. doi: 10.1016/j.mad.2018.12.003, PMID: 30633899

